# Mobile Social Network–Based Smoking Cessation Intervention for Chinese Male Smokers: Pilot Randomized Controlled Trial

**DOI:** 10.2196/17522

**Published:** 2020-10-23

**Authors:** Jinsong Chen, Elsie Ho, Yannan Jiang, Robyn Whittaker, Tingzhong Yang, Christopher Bullen

**Affiliations:** 1 National Institute for Health Innovation School of Population Health University of Auckland Auckland New Zealand; 2 School of Population Health University of Auckland Auckland New Zealand; 3 Centre for Tobacco Control Research Zhejiang University Hangzhou China

**Keywords:** mHealth, mobile smoking cessation, social network-based intervention, smoking cessation, public health, gamified health interventions

## Abstract

**Background:**

Around 2 million Chinese people, mostly men, die annually from tobacco-related diseases; yet, fewer than 8% of Chinese smokers ever receive any smoking cessation support.

**Objective:**

This study aimed to test the preliminary effectiveness and feasibility for a mobile social network (WeChat)–based smoking cessation intervention (SCAMPI program) among Chinese male smokers.

**Methods:**

Chinese male smokers aged 25-44 years were recruited online from WeChat, the most widely used social media platform in China. Individuals using other smoking cessation interventions or who lacked capacity to provide online informed consent were excluded. Participants were randomly assigned (1:1) to intervention or control groups. Neither participants nor researchers were masked to assignment. The trial was fully online. All data were collected via WeChat. The intervention group received access to the full-version SCAMPI program, a Chinese-language smoking cessation program based on the Behaviour Change Wheel framework and relevant cessation guidelines. Specific intervention functions used in the program include: planning to help users make quitting plans, calculator to record quitting benefits, calendar to record progress, gamification to facilitate quitting, information about smoking harms, motivational messages to help users overcome urges, standardized tests for users to assess their levels of nicotine dependence and lung health, as well as a social platform to encourage social support between users. The control group had access to a static WeChat page of contacts for standard smoking cessation care. Both groups received incentive credit payments for participating. The primary outcome was 30-day biochemically verified smoking abstinence at 6 weeks after randomization, with missing data treated as not quitting. Secondary outcomes were other smoking status measures, reduction of cigarette consumption, study feasibility (recruitment and retention rate), and acceptability of and satisfaction with the program.

**Results:**

The program recorded 5736 visitors over a 13-day recruitment period. We recruited 80 participants who were randomly allocated to two arms (n=40 per arm). At 6 weeks, 36 of 40 (90%) intervention participants and 35 of 40 (88%) control participants provided complete self-reported data on their daily smoking status via WeChat. Biochemically verified smoking abstinence at 6 weeks was determined for 10 of 40 (25%) intervention participants and 2 of 40 (5%) control participants (RR=5, 95% CI 1.2-21.4, *P*=.03). In the intervention group, the calculator function, motivational messages, and health tests were underused (less than once per week per users). Participants rated their satisfaction with the intervention program as 4.56 out of 5.00.

**Conclusions:**

Our program is a novel, accessible, and acceptable smoking cessation intervention for Chinese male smokers. A future trial with a greater sample size and longer follow-up will identify if it is as effective as these preliminary data suggest.

**Trial Registration:**

ANZCTR registry, ACTRN12618001089224; https://tinyurl.com/y536n7sx

**International Registered Report Identifier (IRRID):**

RR2-18071

## Introduction

### Problems With Smoking in China

Nearly 300 million people in China smoke tobacco daily [1], 95% of whom are men [1]. Smoking causes 25% of Chinese male deaths [2]. However, tobacco control measures are not well adhered to, and fewer than 8% of Chinese smokers have ever received any smoking cessation advice [3]. Interest in smoking cessation is modest (only 38.8% of all current smokers), and success is rare (10.7% among people who had ever tried to quit smoking) among Chinese male smokers [4].

### Mobile Phone–Based Smoking Cessation Interventions

Mobile phone–based smoking cessation interventions have the potential to address this problem. A Cochrane review of SMS messaging trials for smoking cessation found them to be effective [5]. Apps may also help; in a trial of a behavioral, decision-aid smartphone app, 23.8% of participants self-reported 3-month continuous smoking abstinence compared with 10.2% of participants who did not receive access to the decision-aid app [6].

Smartphone ownership in China is dramatically increasing, especially in the past 5 years [7], to the point where China now has the largest smartphone market (>700 million people) in the world [8]. The largest user group is composed of men aged 25-44 years [9]. Smartphone-based smoking cessation interventions might be a solution to address the problems of low awareness and usage of smoking cessation services in China.

### WeChat Usage

WeChat is the most popular social network platform in China. In 2020, 1.2 billion people were monthly active users of WeChat. WeChat users are highly engaged with the app; nearly 80% of WeChat users use the app for >30 minutes daily [10]. In 2016, WeChat launched a new feature, “mini-program,” to deliver messages, collect user data, and respond to user commands [11]. This makes the WeChat platform more powerful than ever before.

The WeChat super app is becoming a vital component of health care in China [12]. In the past 5 years, a number of studies have attempted to identify the effectiveness and feasibility of delivering health care interventions through WeChat, such as self-management of hypertension or type 2 diabetes [13-16]. Some studies have found improvements in users’ health conditions [15-17]. However, WeChat’s application for smoking cessation remains untapped. Given the large number of WeChat users and the high prevalence of smoking in China, WeChat should be tested to see if it is an appropriate and acceptable platform to deliver smoking cessation interventions.

### Aim

We aimed to evaluate the preliminary effectiveness and feasibility of SCAMPI, a standalone online smoking cessation intervention designed, developed, and trialed entirely within the WeChat ecosystem to reach Chinese men aged 25-44 years who smoke tobacco. 

## Methods

### Study Design

We used a two-arm, parallel, randomized controlled trial design to evaluate preliminary effectiveness and acceptability of the SCAMPI program as well as recruitment and retention for estimating the sample size of a future definitive trial. All trial procedures were conducted online via WeChat. Ethical approvals were granted by The University of Auckland Human Participants Ethics Committee (Ref. 021649) and Zhejiang University School of Public Health Research Ethics Committee (Ref. ZGL201801-2). The trial was prospectively registered with the ANZCTR registry (ACTRN12618001089224).

### Participants

Participants were recruited from China via WeChat advertisements. People interested in participating were invited to complete an electronic questionnaire on WeChat. Inclusion criteria included smokers (daily smokers “smoking any type of tobacco products on a daily basis” or occasional smokers “smoking any type of tobacco products occasionally”) aged 25-44 years; access to a smartphone; knowledge of the Chinese language and WeChat platform; and willingness to participate and provide follow-up information at scheduled points. Participants were excluded if they used other types of smoking cessation interventions, refused to provide follow-up information, or had a medical condition (eg, mental health conditions or cognitive issues that prohibit understanding of the information provided by the program) that could limit their ability to participate. The first 80 eligible individuals who completed questionnaires and subscribed to the SCAMPI Official Account (OA), a social network account used for messaging and interacting with other subscribers, were consented, enrolled in the trial, and randomized. We collected consent via WeChat; then, we asked participants to provide data on demographics, smoking status, acceptability, and satisfaction (intervention group only) with the program via online questionnaires. Participants’ use patterns and engagement with the program were collected via system records of interaction.

### Randomization and Masking

Participants were randomly allocated into intervention and control groups at a 1:1 ratio using a computer-generated randomization sequence with variable block sizes of 2 or 4, provided by the trial statistician (YJ). Participants enrolled themselves to the trial by providing informed consent, completing the registration questionnaire, and subscribing to the SCAMPI OA. The investigator (JC) assigned participants to the intervention based on the randomization sequence.

Due to the nature of the intervention, participants were not masked to the intervention, as they were provided information about the interventions they would have access to. Participants were not informed which version of the program they used —whether it was the intervention of interest or the comparator. Versions 1 and 2 were used to represent the full and restricted versions of the SCAMPI program, respectively.

Researchers were not masked to the treatment allocation, and statistical analyses were not blinded to the treatment allocation. There are multiple sources used for this trial, and 2 of the sources are only used to collect data (SCAMPI mini-program usage data collected via the mini-program server and users’ satisfaction data collected via online questionnaires) from participants in the intervention group. It is impossible to blind researchers to the treatment allocation in order to export data from these sources.

### Recruitment

The advertisement and recruitment of the study were completely online through the WeChat platform. WeChat users who were interested in quitting smoking and typed keywords like “smoking,” “stop smoking,” and “smoking cessation support” would be able to find the SCAMPI OA from WeChat. WeChat users who subscribed to the SCAMPI OA were asked if they would like to take part in the trial through automated WeChat messages. The message was sent in Chinese, translated as “Would you like to be part of a trial for evaluating the preliminary efficacy and feasibility of a WeChat-based smoking cessation program? Use the following link to register as a participant of the trial!” The Tencent Games Dreaming Plan (a department of the Interactive Entertainment Group) also posted the QR code of the SCAMPI OA in their platform to promote the trial and program. After tapping the link, potential participants were directed to the registration system and asked to provide informed consent (see Multimedia Appendix 1). Participants were expected to provide their consent electronically by tapping the “agree” button on the web page. Participation was completely anonymous. Any data related to the participant’s personal identity were not collected. A simple IP address check was used to ensure people did not use multiple identities (one participant participated the study with different WeChat accounts).

### Procedures

After providing informed consent, participants were randomly allocated to different groups (intervention and control group, n=40 in each group). Participants randomized to the intervention group had access for 6 weeks to the full version of the SCAMPI program, and participants randomized to the control group had access for 6 weeks to a restricted version of the SCAMPI program. On completion of the 6-week follow-up assessment, participants in the control group were offered full access to the SCAMPI program. All data on program use were recorded until the end of the trial. All participation and procedures of the trial were completed online through the WeChat platform, except participants who reported 30-day continuous smoking abstinence at the 6-week follow up received a cotinine test kit via courier to test nicotine levels in their saliva. A link to a courier WeChat mini-program was sent to this group of participants. Participants who received the link were asked to provide their address and recipient details to the courier through WeChat (none of these data were accessible nor recorded by the research team). Based on the details, the kits were sent to the participants. Participants were requested to use the kit as instructed, take a photo or shoot a short video about how they used the kits and the relevant results (personal images were not requested), and send the test results to the SCAMPI OA. All data were collected online via the WeChat platform. Participants in both groups recorded their daily cigarette consumption for the week throughout the trial period. Usage data on the corresponding versions of the SCAMPI program were recorded on the server of the program. Participants’ baseline data and satisfaction with the intervention were collected through electronic questionnaires (registration questionnaire and end-of-trial questionnaire). Compensation for participation was delivered in a form called WeChat red packet, a widely used online currency in China and transferrable through the WeChat platform. Recipients of WeChat red packet can use it to purchase goods or use services in China. WeChat red packet was attached to the registration questionnaire, 6-weekly smoking status check-in sessions, and end-of-trial questionnaire (for the intervention group, this is delivered along with the last weekly smoking status check-in session) in the trial. Once participants complete the questionnaires, the WeChat red packet appears in their account (participants in both groups receive same value red packets). Participants’ answers to questionnaires had no impact on the WeChat red packet they received.

### Interventions

Details of the SCAMPI program and its development have been published elsewhere [18]. In brief, the program was developed through a 1-month collaborative product development process involving 20 Chinese male smokers aged 25-44 years recruited through WeChat. We analyzed their smoking behavior using the “Capability,” “Opportunity,” “Motivation,” “Behaviour” (COM-B) model [19], along with relevant reports of the 2015 Chinese Adult Tobacco Use Research [20] and the national online survey about smoking behaviors of Chinese smokers [21]. The collaborative product development process identified a number of behavioral factors affecting the smoking behavior of the target population, as shown in Textbox 1.

**Summary of behavioral factors for smoking.**

Psychological capability

1. Lack of methods to cope with unwanted emotion
2. Low health literacy of smoking harms and second-hand smoking harms
3. Low health literacy of cessation benefits to self and family
4. Lack of methods to quit smoking
5. Lack of awareness of existing smoking cessation services

Reflective motivation

1. Problematic impression of smoking (eg, smoking is cool)

Automatic motivation

1. Perception of smoking as an emotional coping tool (ie, unwanted emotion triggers smoking behavior)
2. Perception of smoking as an entertainment tool (ie, entertainment, such as playing cards, triggers smoking behavior)

Physical opportunity

1. High probability of being in a triggering environment (eg, gifted with cigarettes, peer smoking)
2. Low cost of tobacco

Social opportunity

1. Using smoking as a social communication tool (eg, peer smoking and sharing cigarettes)

Using the Behaviour Change Wheel framework, interventions were designed to address the behavioral factors identified. These interventions adhered to the China Clinical Smoking Cessation Guideline, were coded as functions in the SCAMPI program [19,22], and were deliverable by the WeChat platform [23,24] (see Multimedia Appendix 2). Participants indicated their preferences for the functions in a WeChat-based smoking cessation intervention via an online questionnaire. Prototype iterations and questions were posted on SCAMPI WeChat OA to solicit participants’ preferences on the user experience and user interfaces. Participants’ comments on the prototype helped developers to iteratively update the SCAMPI program before its launch. At the end of the development phase, the 20 participants were invited to rate usability and acceptability by completing an online questionnaire based on the Mobile App Rating Scale questionnaire [25]. The online questionnaire was completed by 16 of the 20 participants, and the average rating for the SCAMPI program was 4.4 out of 5.0.

The program has the following functions: (1) smoking cessation planning, (2) calculator to record quitting benefits (eg, money saved by not smoking), (3) calendar to record progress of smoking cessation (eg, on which dates did the users not smoke), (4) gamification to facilitate quitting (eg, ranking board for users to compete for the longest continuous smoking abstinence), (5) information about smoking harms and health benefits from smoking cessation, (6) motivational messages (based on requests from users) to help users overcome urges, (7) standardized tests to help users assess their levels of nicotine dependence and lung health, and (8) social support platform to deliver peer support between users. Screenshots and QR codes of the SCAMPI program are provided in Multimedia Appendix 3. At the end of the trial, all WeChat users had free access to the SCAMPI program by either using the keywords “smoking/smoking cessation/SCAMPI” on the WeChat platform or scanning the SCAMPI program QR codes. The full version of the SCAMPI program has the aforementioned functions and content. The restricted version of the program provided the users with contact information for standard smoking cessation care (eg, Quitline in China, local smoking cessation clinics). The minimum frequency of program use is once a week, to provide participants’ daily cigarette smoking status throughout the week. Prompts and messages (eg, motivational messages to support users to avoid smoking urges or prompt users to read program posts about smoking harms) were triggered by requests from users. The only messages users received were reminders to provide their smoking status, triggered in the 48 hours after the date users were requested to provide their daily smoking status for the week. The reminders were delivered <3 times a day (once every 8 hours) for 2 days via WeChat. Participants who did not respond to any of these messages were counted as dropouts from the trial. There was no specific training session provided to users. After successfully registering as participants to the study, participants received a message to introduce the versions of program they would use in the trial. Since the program was built natively on the WeChat platform using the WeChat mini-program design guideline, users were expected to have no challenges with using the functions of the program.

### Outcomes

The primary outcome was biochemically verified past 30-day smoking abstinence at 6 weeks [25]. Quit failure was defined as any number of cigarettes smoked in the past 30 consecutive days. This measure is known as the 10th level of smoking abstinence in the Chinese Standard for Smoking Cessation [24]. Participants who reported themselves as 30-day smoking abstainers at the 6-week follow-up received cotinine test strip kits by post. These participants were requested to complete the test at home (using their saliva, with clear instructions provided) and upload a photo or a short video of the strip test results to the investigators via WeChat.

Secondary outcomes were cigarette consumption, 7-day self-reported smoking abstinence at the 4-week and 6-week follow-ups, self-reported 30-day smoking abstinence at the 6-week follow-up (all measured by online, self-reported data), retention in the trial, number of times the participants interacted with their program throughout the trial, and satisfaction ratings for using SCAMPI as a smoking cessation tool (intervention group only).

We used 2 electronic questionnaires. The registration questionnaire asked participants about their demographics, smoking status, and willingness to quit smoking (see Multimedia Appendix 4). The registration questionnaire was developed based on the national online survey about smoking behaviors of Chinese smokers [20,21]. The end-of-trial questionnaire (see Multimedia Appendix 5) was developed based on a standard questionnaire, the Mobile App Rating Scale questionnaire [26]. It focuses on assessing users’ perception of and satisfaction with the app, software, or mobile program they used. Both questionnaires were reviewed and commended by experts from WeChat to ensure its user experience met the Chinese users’ preferences. Usage was measured by the number of times users visited the program. Participants were encouraged to make comments by directly sending text or audio messages to the program if they wished.

### Guidelines for Participant Withdrawal

Participants were informed in the participant information sheet that they were able to withdraw from the trial at any time with no reasons needed. If a participant reported severe physical or mental discomfort by using the SCAMPI program, the investigator (JC) terminated his participation immediately and referred him to health care professionals. Events were recorded and reported.

### Statistical Analysis

In this pilot trial, we recruited around 15% of the total sample required for a future adequately powered trial (ie, 80 participants, 40 per group). We estimate a full trial would need a total of 530 participants (n=265 per group) for 90% power at 5% significance (2-sided) to detect an absolute difference of 10% in the primary outcome between 2 groups, assuming a control quit rate of 6.7% [4]. The feasibility outcomes (eg, recruitment, retention, participants’ demographics, acceptability) are reported descriptively. All randomized participants were included in the final analysis following the intent-to-treat principle. Missing smoking outcomes were considered as treatment failure and imputed as not quitting. Baseline demographics were summarized by randomized group using descriptive statistics. Primary and secondary outcomes are presented as mean (SD) for continuous variables and n (%) for categorical variables. To test the hypothesis of a difference between the 2 randomized groups after intervention, smoking outcomes were analyzed using a Chi-square test or the Fisher exact test when the counts were <5. Both relative risk (RR) and odds ratio (OR) were estimated with 95% CIs. For continuous outcomes, we used a 2-sample *t* test or Wilcoxon non-parametric test for skewed data. A generalized linear mixed model was used to analyze daily cigarette consumption reported by the participants over the 6-week trial period, using Poisson distribution with a log link. Statistical analysis was performed using SAS version 9.4 (SAS Institute Inc, Cary, NC). All statistical tests were 2-sided at a 5% significance level.

## Results

**Figure 1:**
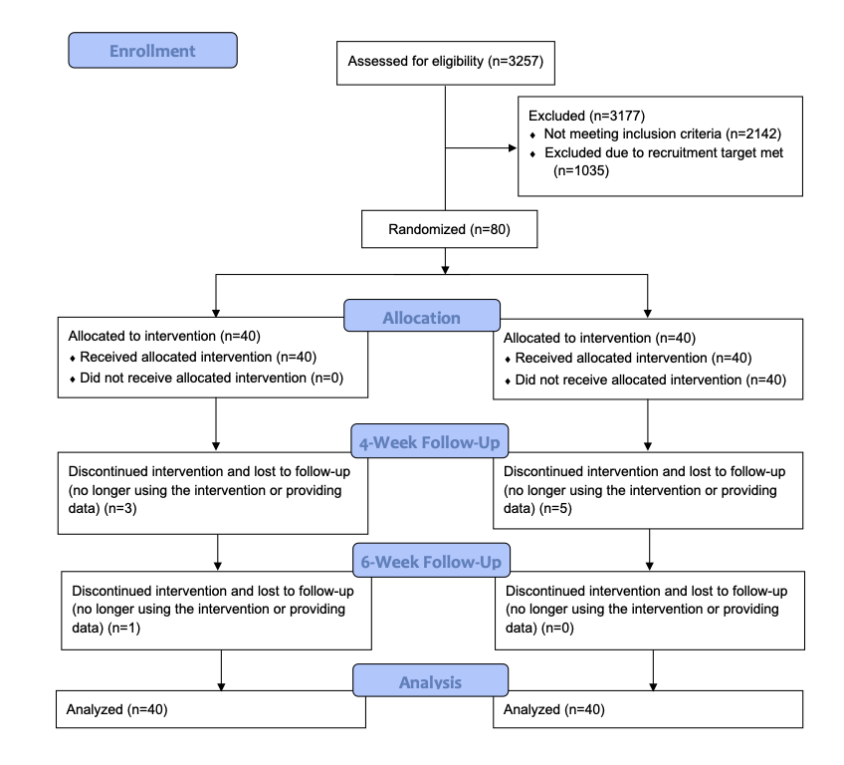
Flowchart of trial recruitment, enrollment, and randomization.

Between January 18, 2019, and January 31, 2019, 80 eligible participants were randomly assigned to the intervention group (n=40) or control group (n=40), as shown in Figure 1.

The intervention and follow-up period lasted for 42 days, from January 31, 2019 to March 13, 2019. In the 13-day recruitment period, 5736 visitors viewed the registration system, 3257 of whom tried to complete the registration form; 1115 eligible individuals provided informed consent and completed the registration forms, among whom the first 80 who completed the registration questionnaire and subscribed to the SCAMPI OA were registered in the study. Table 1 shows the baseline characteristics, smoking status, and quitting reasons of these participants.

**Table 1:** Baseline characteristics, smoking status, and quitting reasons.

Characteristics		Intervention group (n=40)	Control group (n=40)
Age (years), mean (SD)		32.4 (6.0)	31.4 (5.1)
**Smoking status, n (%)**			
	Daily smoker	34 (85)	38 (95)
	Non-daily smoker	6 (15)	2 (5)
**Past smoking cessation attempt, n (5)**			
	Yes	33 (83)	31 (78)
	No	7 (18)	9 (23)
**Use of smoking cessation services, n (%)**			
	Yes	24 (72)	28 (90)
	No	9 (27)	3 (10)
**Factors that trigger smoking, n (%)**			
	Socializing	17 (43)	19 (48)
	At meals	10 (25)	19 (48)
	When feeling down	26 (65)	32 (80)
	When feeling excited	23 (58)	24 (60)
	When nervous and anxious	17 (43)	26 (65)
	When feeling tired	26 (65)	29 (73)
	When involved with entertainment	13 (33)	16 (40)
	When reading or writing	0 (0)	8 (20)
	When working	9 (23)	12 (30)
	When alone	11 (28)	14 (35)
	Drinking alcohol	10 (25)	17 (43)
**Reasons to quit smoking, n (%)**			
	Personal health	20 (50)	21 (53)
	Family health	32 (80)	28 (70)
	Doctor’s advice	16 (40)	18 (45)
	Smoke-free policy in public area	15 (38)	17 (43)
	Social stigma	4 (10)	11 (28)
	Concerned about environment for next generation	9 (23)	17 (43)
**Marital status, n (%)**			
	Single	9 (23)	8 (20)
	In a relationship	9 (23)	6 (15)
	Married	22 (55)	26 (65)
**Parenting status, n (%)**			
	Has a child (or children)	31 (78)	23 (58)
	No child	9 (23)	17 (43)

### Demographics

Of the participants, 90% (72/80) were daily smokers. On average, participants had a strong desire to quit smoking (median quit smoking willingness score=4.3/5.0, interquartile range 4.1-4.5). In addition, 64 of 80 (80%) participants had tried to quit smoking, and 52 of 64 participants (81%) reported having previously used smoking cessation services. Major triggers to smoking were unwanted emotions like feeling depressed and feeling tired as well as concerns for personal or family health.

### Retention

At 6 weeks, 36 of 40 (90%) intervention participants and 35 of 40 (88%) control participants provided complete self-reported data on their daily smoking status via WeChat. For the remaining 9 participants, 7 did not respond, and 2 provided partial smoking data (both were smokers).

Self-reported 30-day smoking abstinence was then collected from a total of 73 participants. Of these participants, 20 (intervention n=15, control n=5) self-reported quitting at 6 weeks and were asked to provide their addresses to receive cotinine test kits to verify their self-reported data. Of these 20 participants, 17 (85%) provided their addresses, of whom 16 (16/20, 80%, intervention n=13, control n=3) completed the tests.

Data on biochemically verified 30-day smoking abstinence at 6 weeks (primary outcome) was available for 69 participants (intervention n= 35, control n=34). Primary analysis included all 80 participants, with missing outcomes treated as not quitting (ie, treatment failure).

### Primary Outcome

Of the 40 participants in the intervention group, 10 (25%) were abstinent (biochemically verified) at the 6-week follow-up, compared with only 2 (5%) of the 40 participants in the control group (RR=5.0, 95% CI 1.2-21.4; *P*=.03). The unadjusted OR was 6.3 (95% CI 1.3-31.1).

### Secondary Outcomes

Secondary outcomes are summarized in Tables 2 and 3.

**Table 2:** Summary of participation and smoking data.

Outcomes		Intervention group (n=40)		Control group (n=40)	
**Retention rate, n (%)**					
	Week 4		37 (93)		35 (88)
	Week 6		36 (90)		35 (88)
Number of times interacting with the SCAMPI program during week 6, mean (SD)		81.88 (37.23)		61.40 (26.40)	
**Cigarettes smoked per day, mean (SD)**					
	Week 4		3.43 (6.39)		7.60 (8.79)
	Week 6		3.00 (6.02)		5.81 (7.72)

**Table 3:** Odds ratios for secondary outcomes.

Outcomes			Intervention group, n (%)		Control group, n (%)		Odds ratio (95% CI)		*P* value
Cigarette consumption reduction at week 6			25 (63)		26 (65)		0.89 (0.36-2.23)		.82
**7-day self-reported smoking abstinence**									
	Week 4	25 (63)		13 (33)		3.46 (1.38-8.69)		.007	
	Week 6	23 (58)		15 (38)		2.25 (0.92-5.52)		.07	
30-day self-reported smoking abstinence at week 6			15 (38)		5 (13)		4.2 (1.35-13.06)		.02

#### Engagement

On average, participants in the intervention group had 82 interactions with their assigned program, while participants in the control group had an average of 61 interactions with their program over the 6-week trial period.

#### Smoking Behavior Change

Both groups reduced cigarette consumption over the 6 weeks, although the average daily consumption was lower in the intervention group, but the difference was not statistically significant (RR=0.47, 95% CI 0.16-1.35; *P*=.16). Compared to baseline, 25 and 26 participants achieved cigarette consumption reduction at week 6 in the intervention and control groups, respectively (unadjusted OR 0.89, 95% CI 0.36-2.23; *P*=.82). At the 4-week follow-up, 25 of 40 participants (63%) in the intervention group and 23 of 40 participants (33%) in the control group reported 7-day smoking abstinence. At week 6, 23 of 40 participants (58%) in the intervention group and 15 of 40 participants (38%) in the control group achieved 7-day smoking abstinence (unadjusted OR at 4 weeks=3.46, 95% CI 1.38-8.69; *P*=.007; unadjusted OR at 6 weeks=2.25, 95% CI 0.92-5.52; *P*=.07). At 6 weeks, 15 of 40 participants (38%) in the intervention group and 5 of 40 participants (13%) in the control group reported 30-day smoking abstinence (RR=3.0, 95% CI 1.2-7.5; unadjusted OR=4.2, 95% CI 1.35-13.06; *P*=.02).

#### Satisfaction

Overall, 36 of 40 participants (90%) from the intervention group responded to the end-of-trial questionnaire, rating the SCAMPI program 4.6 out of 5 (95% CI 4.3-4.8). Almost all of the responding participants (35/36, 97%) were willing to introduce SCAMPI to others, 23 of 36 participants (64%) were willing to pay to use the program, 35 of 36 participants (97%) indicated they would use the program at least once in the next 12 months, and 27 of 36 participants (75%) reported that they would use the program more than 10 times in the next 12 months.

## Discussion

### Principal Findings

In this pilot smoking cessation program underpinned by a theoretical model and empirical evidence [27], Chinese male smokers aged 25-44 years who used the program achieved significantly higher 30-day (both self-reported and biochemically verified) smoking abstinence at the 6-week follow-up as well as 7-day self-reported smoking abstinence (at the 4-week and 6-week follow-ups) than those randomized to the control program. This is the first trial done entirely within the WeChat ecosystem [28-31]. The potential for efficient recruitment (3257 visitors within 13 days) supports the use of this popular social network platform to engage smokers, deliver interventions, and conduct trials via the same platform. Significant numbers of participants (n=52) had ever used smoking cessation services, which may be why the participants engaged with the registration system after reading the advertisement on WeChat. The high retention rate in both arms suggests our strategy of using frequent micro-incentive credits was effective. The program also achieved remarkably high satisfaction ratings from its users.

### Limitations

There were a number of limitations. As a pilot trial, it was not powered to detect a significant effect, and the study period (6 weeks) was insufficient to assess long-term (6-month or 12-month) smoking abstinence. However, this pilot trial has shown that the SCAMPI program is feasible and acceptable as a smoking cessation tool on WeChat, for the largest social network user group and smoking population group in China. The recruitment strategy will be able to recruit a bigger sample size within a reasonable length of time (approximately 1 month). The trial design and mode of intervention delivery have shown the ability to maintain a high retention rate of study participants. A future randomized control trial is needed with a larger sample size and 6-month follow-up to evaluate the program’s effectiveness in supporting long-term smoking cessation. Our findings may not be generalizable to other people, in China or elsewhere. Nevertheless, WeChat is widely used by people of different age groups and in a range of countries [32,33]. However, SCAMPI was developed using a systematic, replicable method and could be used to tailor mobile social network–based smoking cessation interventions to different population groups.

Another limitation is that participants were not blinded. This may lead to type 1 error as participants in the intervention group may tend to report themselves as smoking- abstinent. However, unlike most online trials relying on self-reported data, biochemically verified data was captured. With more resources, a future trial could use more frequent cotinine testing.

There are a number of concerns with using a social network platform to deliver health interventions and implement clinical trials, including reduced experimental control and the risk of reduced reliability [34-38]. However, these concerns may be addressed by improving the quality of the digital trial and health product application. For example, in future trials, more precise technology could be applied to increase study data validity and reliability (eg, using the mobile or computer camera to verify user identities) [39]. On the other hand, there are advantages; digital trials provide a level of anonymity for people unwilling to participate in face-to-face research, typically because of embarrassment and stigma [40-42].

They also overcome logistical barriers for some participants, such as people with physical disability, transport issues, or geographic remoteness [43]. Applying social network platforms to recruit clinical trial participants and deliver trial interventions can facilitate the inclusion of low-incidence or hidden populations, such as young adult smokers [44]. Therefore, the application of a social network platform may reduce the chances of under-reporting and increase the generalizability of the data [40-45].

User experience with the SCAMPI program may have been impacted by the fact that it was embedded within a social network platform. Users may have been disturbed by WeChat messages while using the program. However, a new WeChat feature allows users to temporarily exit from a mini-program to answer WeChat messages. The SCAMPI program was designed to be simple and concise; no function was designed to be used for longer than 1 minute. No session should take >5 minutes of a user’s time. Currently, the program is only able to collect data on the number of times participants interacted with the program. It was difficult to measure the duration of each interaction. Future studies could monitor the duration of user-program interactions to understand user interaction with the program to improve user experience.

### Implications

The greatest strength of our program and pilot trial was the use of an existing, widely used social network platform, ensuring wide reach, efficient recruitment, and frequent engagement. The preliminary effectiveness and acceptability of the SCAMPI program is a useful reference point for a larger definitive trial and to guide the development and deployment of other social network–based health behavior change interventions. If applied as a common service on the WeChat platform, it may be necessary to find nonmonetary incentives to maintain the high engagement, such as enhanced gamification.

## Appendix

Multimedia Appendix 1 Informed consent form.

Multimedia Appendix 2 Conversion of behaviour factors to functions and content of the SCAMPI programme.

Multimedia Appendix 3 Screenshots and QR-codes of the SCAMPI programme.

Multimedia Appendix 4 Registration questionnaire.

Multimedia Appendix 5 End-of-trial questionnaire.

Multimedia Appendix 6 CONSORT-EHEALTH (V 1.6.1) checklist.

## Abbreviations

COM-B: “Capability,” “Opportunity,” “Motivation,” “Behaviour”
OA: official account
OR: odds ratio
RR: relative risk
