# Supplementary material for: Mobile Social Network–Based Smoking Cessation Intervention for Chinese Male Smokers: Pilot Randomized Controlled Trial
Source: JMIR Mhealth Uhealth. 2020 Oct 23;8(10):e17522. doi: 10.2196/17522 (PMC7647814; doi:10.2196/17522)
Supplement: Multimedia Appendix 1 [file mhealth_v8i10e17522_app1.docx]

Multimedia Appendix 1: Informed consent form

**注册参与（SCAMPI）小龙虾戒烟项目**

你好，这是腾讯合作学术机构奥克兰大学的科研调查。该项目的主要意图在于通过一个随机对照组实验来探究（SCAMPI）小龙虾戒烟程序的有效性，本次问卷样本选取为男性样本。

如果你符合参与者条件，欢迎你注册成为此项目的一员。每周你将会获得微信红包。
注：
1. 完成答卷并回复“完成”到公众号“NIHISCAMPI”注册成为项目参与者。未回复公众号的答卷将视为无效答卷，敬请谅解。
2. 参与过程中请勿修改微信名称，如若修改，请告知公众号“NIHISCAMPI”。

**参与者条件：**
- 中国男性烟民（年龄25 ～ 44岁）
- 有强烈意愿戒烟

**参与项目包括 ：**
- 填写信息
- 使用SCAMPI小程序
- 接收吸烟危害的信息
- 吸烟状况输入后参与红包抽奖（每周一次，总计35元）
- 非实验室唾液测试（仅需提供照片或小视频）
- 填写程序评测问卷

**安全与信息：**
此项目不会对你的身心照成任何情况的影响，如果让你觉得有任何不适，可联系：
浙江大学医学部烟草控制研究中心
地址：浙江大学紫荆港校区25号楼
电话：0571-88208219
所有个人信息都将被做保密处理并不会暴露在任何与研究相关的发表物中。

**项目结果：**
我们将在2019年的4月份完成所有数据的收集，数据分析的相关发现有可能发表于学术论文中，我们将在SCAMPI公众号中提供相关链接，参与者可以随时进行查阅。

**联系方式：**
陈锦松（博士研究生-新西兰奥克兰大学）
邮件：jinsong.chen@auckland.ac.nz
如若有其他相关的道德伦理问题，欢迎你联系:
新西兰奥克兰大学人伦道德委员会（021649号）
奥克兰大学，科研办公室
邮件：ro-ethics@auckland.ac.nz
浙江大学公共卫生学院医学伦理委员会（ZGL201801-2号）

**相关条款：**
如果你同意以下条款并确认参与，你可以使用截屏的方式保存这份文件。
- 我同意参与本次科研项目。
- 我已被知会我可以在任何时候以任何理由退出项目。在我退出后，除我已输入的信息外，项目组将不会向我收集任何信息，我也同时失去获取相关奖励的权利。
- 我将会被询问与吸烟行为相关的信息，并每周输入吸烟数据。
- 我已被知会任何与我个人信息相关的数据将不会被收集以及披露。

**注意：**
本活动由新西兰国家健康创新研究院独立发起，由其承担本活动的全部法律责任。腾讯并非该活动的组织方与执行方，不因此承担任何法律责任。

**01**我已经阅读参与者须知以及参与者同意书，并同意参与这个科研项目。*

同意

不同意

**Register and participate the SCAMPI study**

Hello, this is a research project run by Tencent and the University of Auckland. The main intention of this study is to identify the preliminary effectiveness of the SCAMPI programme (a smoking cessation intervention) through a pilot randomised controlled trial. This study will focus on male smokers only.

If you meet the participants requirements, welcome to be one of the study participants. Your compensation for participation will be released as WeChat Red Packet in a weekly basis.

Note:
1. Please send “complete” to the SCAMPI official account “NIHISCAMPI” after you are finishing the register questionnaire, or your questionnaire will be deleted.
2. Please don’t change your WeChat account name during the trial period, or please inform the SCAMPI official account “NIHISCAMPI” if you want to change your WeChat account name.

**Participants requirements:**

-  Chinese male smokers aged 25 to 44 years

-  Have strong intention to quit smoking

**Your involvement:**

-  Fill questionnaire

-  Using the SCAMPI programme

-  Receiving quitting information

-  Providing smoking status to receive red packet compensation (once a week, total value ¥35 RMB)

-  Out of lab saliva testing (you will be requested to provide a photograph or shot video)

-  Fill an end-of-trial questionnaire

**Safety and information:**

We do not anticipate any risks to participants in this project. However, if you experience discomfort, please contact:
Zhejiang University Centre for Tobacco Control Research
Address: No.25 Zijing Gang Campus, Zhejiang University

Contact: 0571-88208219
Website: www.tfcampuschina.com
All information collected from this project will not contain any identifying information. If any, they will be anonymised. When the results of this project are written up or reported in any context, no material that could personally identify you will be used.

**Project results:**

The project will be run over a period until April 2019. We anticipate that the results will be available and published on relevant academic journals. We will also provide a link to the publication for reviewing and reading on the SCAMPI official account.

**Contact:**

Jinsong Chen
PhD candidate,
National Institute for Health Innovation, The University of Auckland
Email: jinsong.chen@auckland.ac.nz
*This project had been approved by:*The University of Auckland Human Participants Ethics Committee (Reference No.: 021649) Email: [ro-ethics@auckland.ac.nz](mailto:ro-ethics@auckland.ac.nz)

The Zhejiang University School of Population Health Medical Ethics Committee (Reference No.: ZGL201801 - 2)

**Terms and conditions:**

(you can take screenshot to save the information in this form)

- I agree to participate this project
- I have been informed that I can withdraw from the project by anytime I want without any reasons (by then except already saved information, all my participation information will be deleted (although you will lose your opportunity to receive your participating compensation)
- I realise I will be asked about my smoking status (in weekly basis) and information in related to my smoking behaviour
- I know all information about my personal identity will not be collected or published

Note:
This project is run by the National Institute of Health Innovation, which is responding to all legal responsibility. Tencent is not the organiser nor operator. Tencent will not take any legal responsibility in regarding to this project.

1. I had read and understand all information about, I agree to take part in this research project.

Agree

Disagree
