# Supplementary material for: Mobile Social Network–Based Smoking Cessation Intervention for Chinese Male Smokers: Pilot Randomized Controlled Trial
Source: JMIR Mhealth Uhealth. 2020 Oct 23;8(10):e17522. doi: 10.2196/17522 (PMC7647814; doi:10.2196/17522)
Supplement: Multimedia Appendix 2 [file mhealth_v8i10e17522_app2.docx]

Multimedia Appendix 2: Conversion of behaviour factors to functions and content of the SCAMPI programme

| **COM-B components** | **BCTs code** | **Description and examples on SCAMPI** | **1** | **2** |
| --- | --- | --- | --- | --- |
| Psychological Capability | BM1 | Provide information on consequences of smoking and smoking cessation  E.g. messages of smoking harms | √ | √ |
|  | BM2 | Boost motivation and self-efficacy  E.g. motivating messages when users about to have a slip of relapse | √ | √ |
|  | BM3 | Provide feedback on current behaviour  E.g. remind users how many days they had achieved smoke free | √ | √ |
|  | BM4 | Provide rewards contingent on successfully stopping smoking  E.g. praise users when he achieved one-week smoke free | √ | √ |
|  | BM5 | Provide normative information about others' behaviour and experiences  E.g. smoke free board shows how others are doing | √ | √ |
|  | BM10 | Explain the importance of abrupt cessation  E.g. provide information about even one cigarette harms | √ | √ |
|  | BS1 | Facilitate barrier identification and problem solving  E.g. provide advice to help users overcome unwanted emotion which is considered as one key factor to trigger smoking | √ | √ |
|  | BS2 | Facilitate relapse prevention and coping  E.g. provide information about how lapses occur and how they lead to relapse and to develop specific strategies for preventing lapses | √ | √ |
|  | BS3 | Facilitate action planning/develop treatment plan  E.g. the programme works with users to generate clear quit plans | √ | √ |
|  | BS4 | Facilitate goal setting  E.g. “30-day smoke free challenge” - quit date and goals that support the aim of remaining abstinent | √ | √ |
|  | BS5 | Prompt review of goals  E.g. prompt users to review how far the he has achieved the goal of abstinence | √ | √ |
|  | BS6 | Prompt self-recording  E.g. the programme provides function for users to record and review potentially useful information | √ | √ |
|  | BS9 | Set graded tasks  E.g. from providing daily smoke free data to taking 30-day smoke free challenge | √ | √ |
|  | BS10 | Advise on conserving mental resources  E.g. advise on ways of minimising stress and access to relevant resources (focus on avoiding unwanted emotions) | √ | √ |
|  | A5 | Give options for additional and later support  E.g. provide links to other smoking cessation services those were available | √ | √ |
|  | RC2 | Elicit and answer questions  E.g. the programme allows users to ask questions whenever they want and aims to answer as soon as possible | √ | √ |
|  | RC5 | Offer/direct towards appropriate written materials  E.g. materials and information shown on the programme will be clearly identified their sources | √ | √ |
|  | RC6 | Provide information on withdrawal symptoms  E.g. information about what are, and are not, nicotine withdrawal symptoms, and so on will be provided | √ | √ |
|  | RC9 | Summarise information/confirm client decisions  E.g. terms and conditions, as well as participation information sheet will be provided before users participate and use the SCAMPI programme | √ | √ |
|  | RC10 | Provide reassurance  E.g. provide information about users’ experience are normal and expected, positive feedback to encourage continual abstinence after using the programme | √ | √ |
| Reflective Motivation | BM6 | Prompt commitment from the client there and then  E.g. users need to assure that if they want to take the challenge of being smoke free for period of time | √ | √ |
|  | BM7 | Provide rewards contingent on effort or progress  E.g. programme gives praise for the effort and progress the user is making toward their goal of abstinence | √ | √ |
|  | BM8 | Strengthen ex-smoker identity  E.g. provide information about the importance of regarding smoking as something that is ‘not an option’, including the ‘not a puff’ | √ | √ |
|  | BM9 | Identify reasons for wanting and not wanting to stop smoking  E.g. this had been accomplished in development questionnaire 1 by identifying reasons of quitting and factors that may prevent successful cessation | √ | √ |
|  | BM11 | Measure CO  E.g. this is not deliverable by the nature of mHealth app-based smoking cessation interventions | √ | X |
|  | RC8 | Elicit client views  E.g. this had also been achieved by development questionnaire 1 by asking users’ view on smoking and smoking cessation, as well as their experience on other cessation services | √ | √ |
| Automatic Motivation | BS10 | Advise on conserving mental resources  E.g. provide advice on methods to minimise stress and access to other resources for mental health services (focus on avoiding using smoking as a method to cope from unwanted emotions) | √ | √ |
| Physical Opportunity | BS7 | Advise on changing routine  E.g. provide advice on changing routine to minimise exposure to smoking cues | √ | √ |
|  | BS8 | Advise on environmental restructuring  E.g. provide advice on ways of changing the physical environment to minimise exposure to smoking cues | √ | √ |
|  | A1 | Advise on stop-smoking medication | NA | NA |
|  | A3 | Adopt appropriate local procedures to enable clients to obtain free medication | NA | NA |
|  | A4 | Ask about experience of stop smoking medication that the smokers is using | NA | NA |
| Social Opportunity | BS11 | Advise on avoiding social cues for smoking  E.g. provide advice on encouraging smoking peer to quit smoking together to minimise chance of exposing to peer smoking situation | √ | √ |
|  | A2 | Advise on/facilitate use of social support  E.g. virtual peer support group will be built on the programme to encourage communication and peer support between programme users | √ | √ |
| Programme Design & Development | RD1 | Tailor interactions appropriately  E.g. information provided by users (e.g. cost of cigarettes smoke) will be used to tailor intervention (cost saved from not smoking) | √ | √ |
|  | RD2 | Emphasise choice  E.g. provide evidence and practice-based choices for users to adapt to keep themselves away from smoking | √ | √ |
|  | RI1 | Assess current and past smoking behaviour  E.g. users’ smoking behaviour will be monitored before, during and after using the programme | √ | √ |
|  | RI2 | Assess current readiness and ability to quit  E.g. users’ readiness and ability to quit will be assessed before using the programme | √ | √ |
|  | RI3 | Assess past history of quit attempts  E.g. users’ history of quit attempts will be assessed before using the programme | √ | √ |
|  | RI4 | Assess withdrawal symptoms  E.g. users will be prompted to report their withdrawal symptoms once they have any during using the programme | √ | √ |
|  | RC1 | Build general rapport  E.g. users mother language (Chinese) will be used and used in a comfortable and manner concerned way to ensure the development of friendly and professional relationship built between the programme and users | √ | √ |
|  | RC3 | Explain the purpose of CO monitoring  Since it is undeliverable by mHealth app-based intervention, the BCT is not applicable to SCAMPI | NA | NA |
|  | RC4 | Explain expectations regarding treatment programme  E.g. information about the programme, what it involves, and what it requires of the users will be clearly indicated before users using it | √ | √ |
|  | RC7 | Use reflective listening  E.g. users will be allowed to communicate with the programme whenever they want, some pre-set responses will be provided for general questions, while manual supports will be provided for specific issues | √ | √ |

BM: specific focus on behaviour (B) and addressing motivation (M)

BS: specific focus on behaviour (B) and maximising self-regulatory capacity/skills (S)

A: promote adjuvant activities (A)

RD: general aspects of the interaction (R) focusing on delivery of the intervention (D)

RI: general aspects of the interaction (R) focusing on information gathering (I)

RC: general aspects of the interaction (R) focusing on general communication (C)

1: China Clinical Smoking Cessation Guideline

2: WeChat mini-programme design guideline

NA: Not Applicable
