# Supplementary material for: Mobile Social Network–Based Smoking Cessation Intervention for Chinese Male Smokers: Pilot Randomized Controlled Trial
Source: JMIR Mhealth Uhealth. 2020 Oct 23;8(10):e17522. doi: 10.2196/17522 (PMC7647814; doi:10.2196/17522)
Supplement: Multimedia Appendix 3 [file mhealth_v8i10e17522_app3.docx]

Multimedia Appendix 3: Screenshots and QR-codes of the SCAMPI programme

| 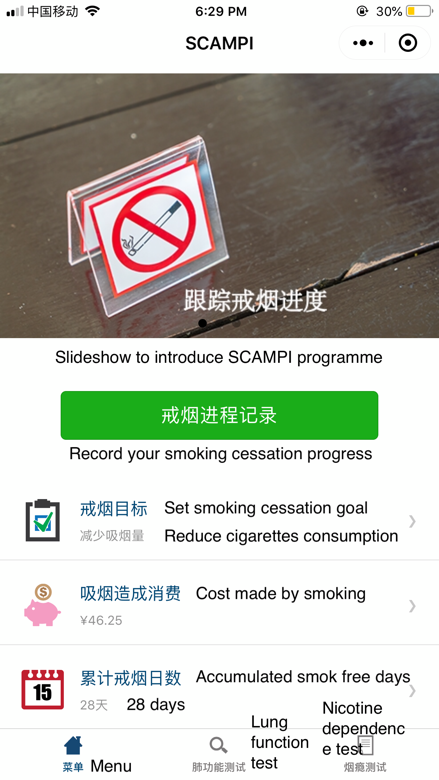 | 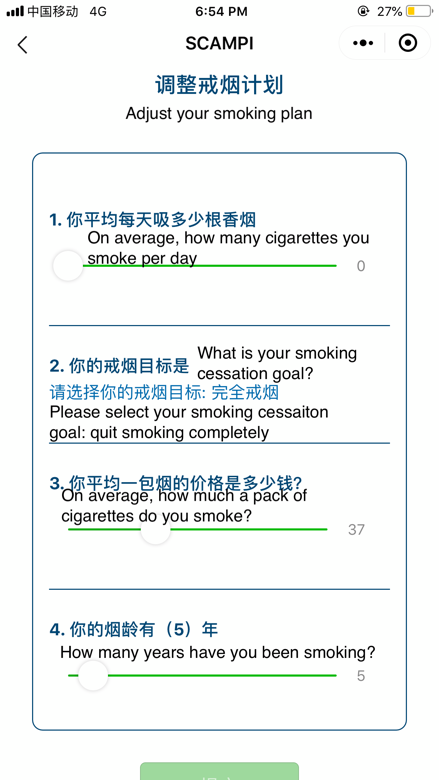 | 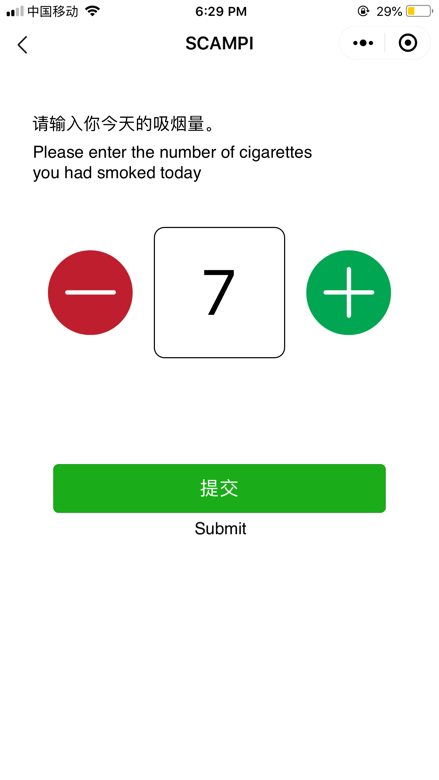 | 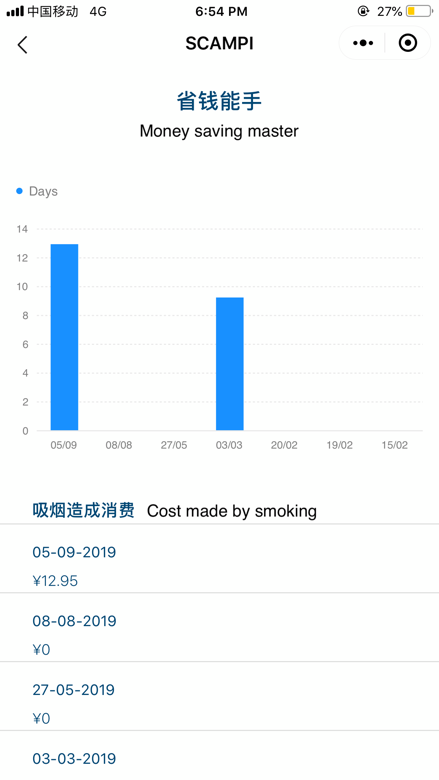 |
| --- | --- | --- | --- |
| Menu of SCAMPI programme | Interface to make smoking cessation plan | Interface to enter daily cigarette consumption | Calculator to record money saved from stopping smoking |
| 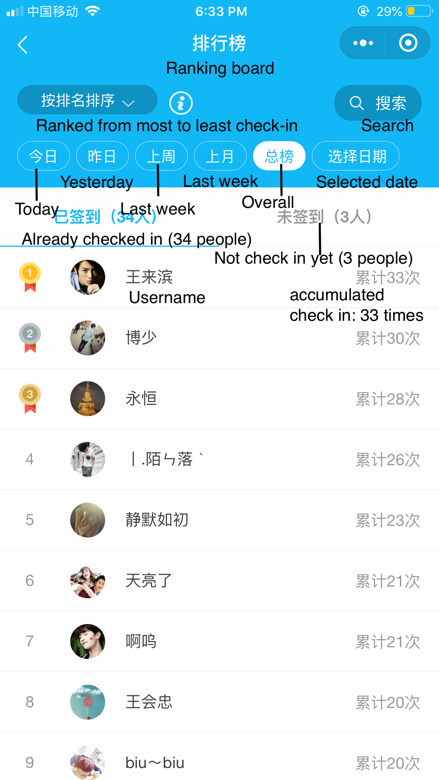 | 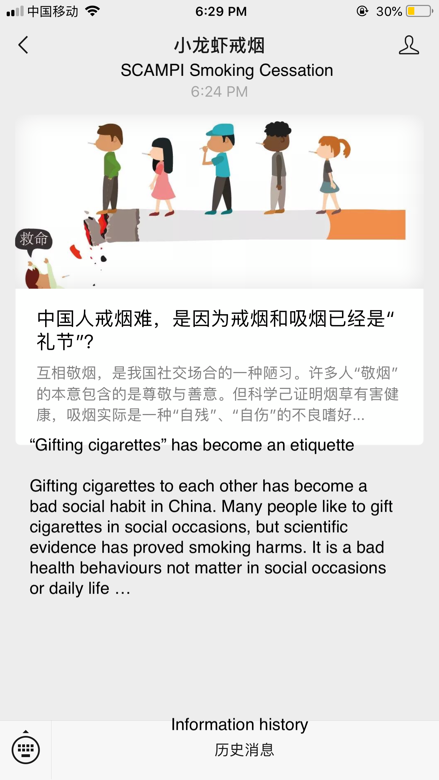 | 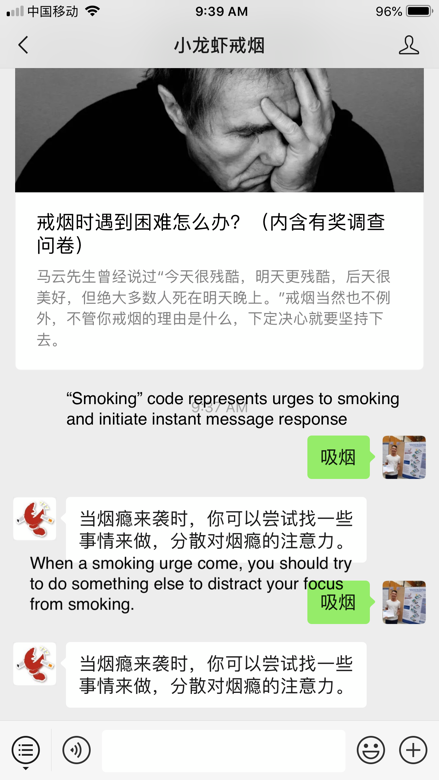 | 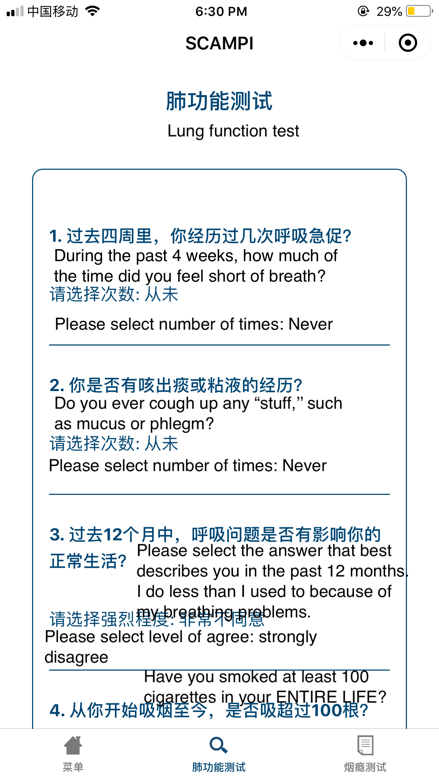 |
| Interface of ranking board | Interface about sending users information of smoking harms | Instant message to support user who have smoking urge | Interface provides standard test to users |
| 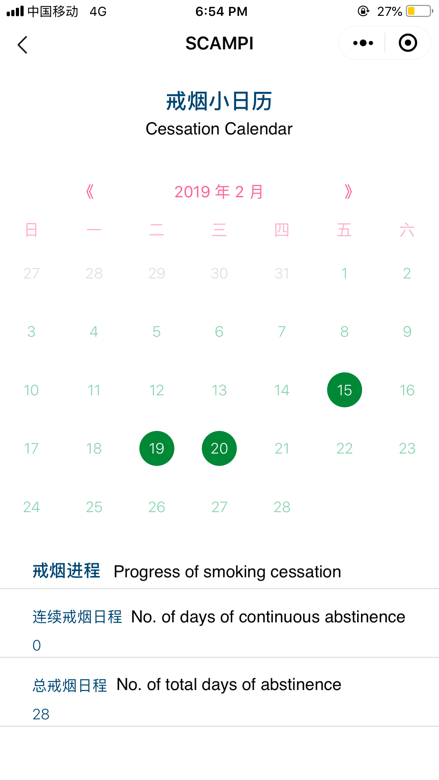 | 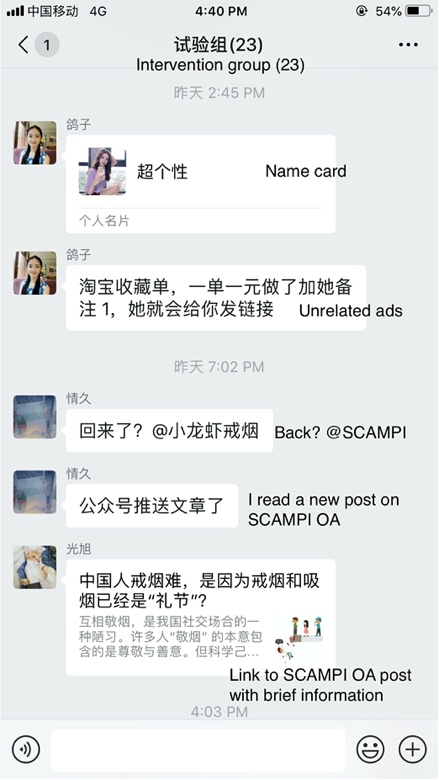 | 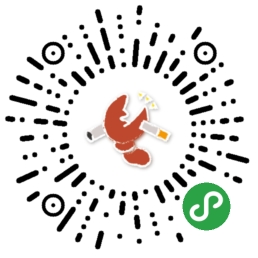 | 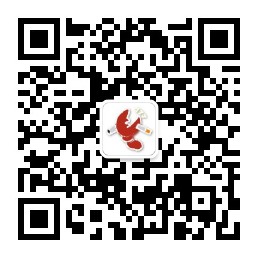 |
| Calendar to record smoking cessation progress | Interface of providing group supports | QR-code to SCAMPI mini-programme | QR-code to SCAMPI official account |
