# Supplementary material for: Mobile Social Network–Based Smoking Cessation Intervention for Chinese Male Smokers: Pilot Randomized Controlled Trial
Source: JMIR Mhealth Uhealth. 2020 Oct 23;8(10):e17522. doi: 10.2196/17522 (PMC7647814; doi:10.2196/17522)
Supplement: Multimedia Appendix 4 [file mhealth_v8i10e17522_app4.docx]

Multimedia Appendix 4: Registration questionnaire

1. Please identify your gender

|  | Male |
| --- | --- |
|  | Female (sorry you did not meet the participant inclusion criteria, you will not be able to take part in this project, thanks for your support) |

2. Which age groups are you belonging in?

|  | 0-24 (sorry you did not meet the participant inclusion criteria, you will not be able to take part in this project, thanks for your support) |
| --- | --- |
|  | 25-44 |
|  | 45-64 (sorry you did not meet the participant inclusion criteria, you will not be able to take part in this project, thanks for your support) |
|  | 65+ (sorry you did not meet the participant inclusion criteria, you will not be able to take part in this project, thanks for your support) |

3. Are you a current smoker?

|  | Yes |
| --- | --- |
|  | No (sorry you did not meet the participant inclusion criteria, you will not be able to take part in this project, thanks for your support) |

4. Do you currently receiving any type of smoking cessation services

|  | Yes (sorry you did not meet the participant inclusion criteria, you will not be able to take part in this project, thanks for your support) |
| --- | --- |
|  | No |

5. Please identify your willingness to quit smoking

|  | 1 (totally unwilling to quit) |
| --- | --- |
|  | 2 |
|  | 3 |
|  | 4 |
|  | 5 (strongly willing to quit) |

**General Information**

6. You started smoking when you were

|  | Under 18 |
| --- | --- |
|  | 18~25 years |
|  | 26~30 years |
|  | 31~40 years |
|  | 41~44 years |

7. Are you a daily smoker?

|  | Yes (to Question 9) |
| --- | --- |
|  | No (to Question 10) |

8. On average, how many cigarettes you smoke per day? (to question 11)

|  | 1-5 stick(s) |
| --- | --- |
|  | 6-10 sticks |
|  | 11-15 sticks |
|  | 16-20 sticks |
|  | 21-25 sticks |
|  | 26-30 sticks |
|  | 31-35 sticks |
|  | 36-40 sticks |
|  | More than 40 sticks |

9. How often do you smoke?

|  | Once every two days |
| --- | --- |
|  | Once every three days |
|  | Once every four days |
|  | Once every five days |
|  | Once every six days |
|  | Once every seven days |

10. Have you tried to quit smoking?

|  | Yes (to Question 12) |
| --- | --- |
|  | No (to Question 17) |

11. How many times have you tried to quit smoking?

|  | Once |
| --- | --- |
|  | Twice |
|  | Three times |
|  | Four times |
|  | Five times |
|  | More than 5 times |

12. The longest time you had stopped smoking lasted for ____month(s)

|  | Less than 1 month (please specify how many days) |
| --- | --- |
|  | 1-3 months |
|  | 4-6 months |
|  | 7-9 months |
|  | 10-12 months |
|  | More than 12 months |

13. Have you ever used / currently using any stop smoking apps?

|  | Yes (to Question 16) |
| --- | --- |
|  | No (to Question 18) |

14. Which types of the following smoking cessation service had you received? (MCQ)

|  | Smoking cessation clinic |
| --- | --- |
|  | QuitLine |
|  | Smoking cessation medication |
|  | Smoking cessation text messaging services |
|  | Smoking cessation apps |
|  | Other (please specify) |

15. Do you think the smoking cessation services you received are helpful?

|  | 1 not helpful at all |
| --- | --- |
|  | 2 |
|  | 3 |
|  | 4 |
|  | 5 very helpful |

16. Why don’t you use smoking cessation services (MCQ)

|  | Did not know mobile smoking cessation apps were available |
| --- | --- |
|  | Do not feel mobile smoking cessation apps are useful |
|  | Never thought about it |
|  | Other reasons (please specify) |

17. Please identify the main factors that usually trigger you to smoke (MCQ)

|  | In social situations (in workplace or business situations with friends, colleagues, etc.) |
| --- | --- |
|  | At (or after) a meal |
|  | Feeling depressed or down in mood |
|  | Feeling positive or happy |
|  | Feeling stressed or anxious |
|  | Feeling tired |
|  | During entertainment (e.g. playing cards, Majiang, watching sports, etc.) |
|  | Reading or Writing |
|  | At work |
|  | When alone |
|  | When drinking alcohol |
|  | Other situations |

18. Please identify the main factors that could motivate you to quit. (MCQ)

|  | Personal health concerns |
| --- | --- |
|  | Family health concerns |
|  | High cost of cigarettes |
|  | Advice and examples from family / friends |
|  | Advice from doctors or other health professionals |
|  | Restrictions on smoking (in work place, on public transportation, at home) |
|  | Social stigma of smoking |
|  | Don’t want child / children growing up with smoking |
|  | Others (please specify) |

19. Your year of birth is:

1974 ~ 1993

20. Please select the city you currently live in:

21. Your current occupation is:

|  | Student |
| --- | --- |
|  | Government worker |
|  | Businessman (office worker) |
|  | Professional (doctor/lawyer/journalist/teacher etc.) |
|  | Worker (factory worker/labour worker etc.) |
|  | Sales and services |
|  | Self-business owner |
|  | Freelancer |
|  | Farmer |
|  | Retired |
|  | Unemployed |
|  | Other (Please specify) |

22. Your current marital status is:

|  | Single |
| --- | --- |
|  | In a relationship |
|  | Married |

23. Do you have child / children

|  | Yes |
| --- | --- |
|  | No |
