# Supplementary material for: Mobile Social Network–Based Smoking Cessation Intervention for Chinese Male Smokers: Pilot Randomized Controlled Trial
Source: JMIR Mhealth Uhealth. 2020 Oct 23;8(10):e17522. doi: 10.2196/17522 (PMC7647814; doi:10.2196/17522)
Supplement: Multimedia Appendix 5 [file mhealth_v8i10e17522_app5.docx]

Multimedia Appendix 5: End-of-trial questionnaire

**End-of-trial Questionnaire**

1. Do you feel the app is interesting?

|  | Not interesting at all |
| --- | --- |
|  | Mostly uninteresting |
|  | OK, would like to use for a brief time (< 5 minutes) |
|  | Moderately interesting; would like to use 5-10 minutes |
|  | Very interesting, would like to use repeatedly |

2. Please let us know if the app will attract Chinese smokers (content, design and functions)?

|  | Not at all |
| --- | --- |
|  | Maybe not |
|  | Not sure |
|  | Maybe yes |
|  | Of course, yes |

3. Do you think the current prototype of the smoking cessation app has functions suitable to you?

|  | Not suitable at all |
| --- | --- |
|  | Partly not suitable |
|  | Neither suitable nor not suitable |
|  | Mostly suitable |
|  | Completely suitable |

4. Do you feel the description of the app is easy to understand (including its icons, menu and instructions)?

|  | Not understandable or followable at all |
| --- | --- |
|  | Somewhat not understandable and followable (takes me more than half an hour to understand and follow) |
|  | Somewhat understandable and followable (takes me 15 minutes to half an hour to understand and follow |
|  | Easy to understand and follow (takes me 5 to 15 minutes to understand and follow) |
|  | The app is very intuitive (I don't need to spend any time to understand and follow) |

5. Do you think the flow of the app makes sense (the transitions between functions or interfaces are clear and logical)?

|  | Very unclear (I cannot see any logic at all) |
| --- | --- |
|  | Somewhat unclear (takes me more than half an hour to understand the logic) |
|  | Somewhat clear (takes me 15 minutes to half an hour to understand the logic) |
|  | Clear (takes me 5 to 15 mins to understand the logic) |
|  | Very clear (I don't need to spend any time to understand the logic) |

6. Do you feel the app prototype has the visually appropriate layout (including the size and location of icons, buttons and menu)?

|  | The layout is very bad, cannot understand at all |
| --- | --- |
|  | The layout is somewhat bad, it’s very hard to understand |
|  | The layout is neither bad nor good |
|  | The layout is somewhat good, able to understand |
|  | The layout is very good, lean and intuitive, can be understood easily |

7. How good does the app look?

|  | Very bad, unpleasant to look at, poorly designed, clashing, mismatched colours |
| --- | --- |
|  | Somewhat bad, poorly designed, bad use of colour, visually boring |
|  | Neither pleasant, nor unpleasant |
|  | Somewhat good, pleasant, seamless graphics, consistent and professionally designed |
|  | Very good, beautiful, very attractive, memorable, stands out, use of colour enhances app features/menus |

8. Would you recommend this app to others?

|  | Not at all, I would not recommend this app to anyone |
| --- | --- |
|  | Mostly not, there are very few people I would recommend this app to |
|  | Not sure |
|  | Mostly yes, there are many people I would recommend this app to |
|  | Definitely, I would recommend this app to everyone |

9. Will you use this app in the next year (about how many times)?

|  | None |
| --- | --- |
|  | 1-2 time(s) |
|  | 3-10 times |
|  | 11-50 times |
|  | more than 50 times |

10. Would you willing to pay for using this app?

|  | Definitely not |
| --- | --- |
|  | Very unlikely |
|  | Possibly |
|  | Very likely |
|  | Definitely yes |

11. Please rate this app.

|  | 1 (worst) |
| --- | --- |
|  | 2 |
|  | 3 |
|  | 4 |
|  | 5 (best) |

12. Please rank the app based on your perception of its effectiveness (1 represent strongly disagree, 5 represent strongly agree)

| Questions | 1 | 2 | 3 | 4 | 5 | Average |
| --- | --- | --- | --- | --- | --- | --- |
| This app has increased my awareness of the importance of quitting smoking |  |  |  |  |  |  |
| This app has increased my knowledge/understanding of the need of quitting smoking |  |  |  |  |  |  |
| The app has positively changed my attitudes toward quitting smoking |  |  |  |  |  |  |
| The app has increased my intentions to stop smoking |  |  |  |  |  |  |
| This app would encourage me to seek further help to quit smoking (if I needed it) |  |  |  |  |  |  |

13. Do you have any further comments about the app, please specify
